# LipidFinder 2.0: advanced informatics pipeline for lipidomics discovery applications

**DOI:** 10.1093/bioinformatics/btaa856

**Published:** 2020-12-10

**Authors:** Jorge Alvarez-Jarreta, Patricia R S Rodrigues, Eoin Fahy, Anne O’Connor, Anna Price, Caroline Gaud, Simon Andrews, Paul Benton, Gary Siuzdak, Jade I Hawksworth, Maria Valdivia-Garcia, Stuart M Allen, Valerie B O’Donnell

**Affiliations:** School of Medicine, Systems Immunity Research Institute, School of Medicine, Cardiff University, Cardiff CF14 4XN, UK; European Molecular Biology Laboratory, European Bioinformatics Institute (EMBL-EBI), Wellcome Genome Campus, Hinxton CB10 1SD, UK; School of Medicine, Systems Immunity Research Institute, School of Medicine, Cardiff University, Cardiff CF14 4XN, UK; Department of Bioengineering, University of California, San Diego, CA 92037, USA; School of Medicine, Systems Immunity Research Institute, School of Medicine, Cardiff University, Cardiff CF14 4XN, UK; School of Biosciences, Cardiff University, Cardiff CF10 3AX, UK; Bioinformatics, Babraham Institute, Cambridge CF24 3AA, UK; Bioinformatics, Babraham Institute, Cambridge CF24 3AA, UK; The Scripps Research Institute, Center for Metabolomics, La Jolla, CA 92037, USA; The Scripps Research Institute, Center for Metabolomics, La Jolla, CA 92037, USA; School of Medicine, Systems Immunity Research Institute, School of Medicine, Cardiff University, Cardiff CF14 4XN, UK; School of Medicine, Systems Immunity Research Institute, School of Medicine, Cardiff University, Cardiff CF14 4XN, UK; School of Computer Science and Informatics, Cardiff University, Cardiff CF24 3AA, UK; School of Medicine, Systems Immunity Research Institute, School of Medicine, Cardiff University, Cardiff CF14 4XN, UK

## Abstract

**Summary:**

We present LipidFinder 2.0, incorporating four new modules that apply artefact filters, remove lipid and contaminant stacks, in-source fragments and salt clusters, and a new isotope deletion method which is significantly more sensitive than available open-access alternatives. We also incorporate a novel false discovery rate method, utilizing a target–decoy strategy, which allows users to assess data quality. A renewed lipid profiling method is introduced which searches three different databases from LIPID MAPS and returns bulk lipid structures only, and a lipid category scatter plot with color blind friendly pallet. An API interface with XCMS Online is made available on LipidFinder’s online version. We show using real data that LipidFinder 2.0 provides a significant improvement over non-lipid metabolite filtering and lipid profiling, compared to available tools.

**Availability and implementation:**

LipidFinder 2.0 is freely available at https://github.com/ODonnell-Lipidomics/LipidFinder and http://lipidmaps.org/resources/tools/lipidfinder.

**Supplementary information:**

Supplementary data are available at *Bioinformatics* online.

## 1 Introduction

Lipidomics describes the discovery and analysis of lipids (fats), which are essential molecules for life in all organisms (Wenk, 2005). However, it is hampered by the lack of specifically tailored informatics tools that effectively clean up raw datasets, which can contain huge numbers of artefacts (around 90–95% of initial signals) as described in detail in our Supplementary Information. Informatics tools used for lipidomics have been to a large extent designed for global metabolomics (and some are still focused primarily on this). The lack of specialized filters for lipidomics can have a negative effect on the output’s robustness since lipids have unique analytical challenges (Cappadona *et al.*, 2012; O'Donnell et al., 2014). In 2017, we published a Python tool, LipidFinder, designed to be an additional stage of the lipidomics pipeline (O'Connor *et al.*, 2017). Specifically tailored for high-resolution LC/MS, LipidFinder processes the output of pre-processing tools, such as XCMS, to filter out common artefacts, including well known ESI contaminants and adducts, and remove background effects. LipidFinder was found to retain most reference lipids, improving the quality of lipidomic data. However, its output datasets still contained a significant level of artefacts, creating substantial problems for downstream statistical power. Thus, we developed new features and here we present LipidFinder 2.0, which integrates a new user-friendly interface to configure its parameters, additional filters and methods to improve the reliability and speed of the output.

A significant issue in identification is over-annotation where MS is used to assign a fully annotated structure. This has led to major mistakes in structural assignment (Bowden *et al.*, 2017; Liebisch *et al.*, 2015). This problem is unique to lipids due to the large numbers of isobaric ions. Currently open-access lipidomics software do not address this. Here, we include an enhanced putative identification procedure that allows different degrees of profiling, returning only the bulk structure. We introduce a novel false discovery rate (FDR) method, using a target–decoy strategy. LipidFinder 2.0 supports as input datasets from popular pre-processing tools. We tested performance with real data, using XCMS-based lipidomics pipeline with/without LipidFinder and results are shown in Supplementary Results.

## 2 System and methods

The lipidomics analysis pipeline including LipidFinder 2.0 is outlined inFigure 1. Major changes have been introduced in its internal framework to improve usability, performance and reliability. These are explained in detail in the Supplementary Information and summarized here.

**Fig. 1.**
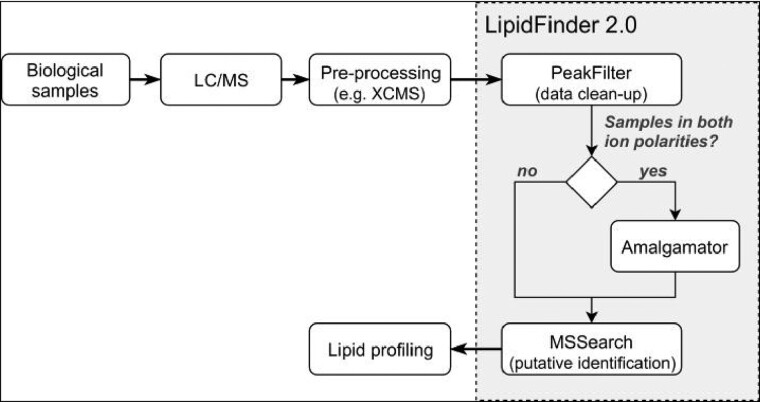
Common pipeline for untargeted lipidomics incorporating LipidFinder 2.0. It also shows LipidFinder’s new main workflow

LipidFinder is designed to be integrated after the pre-processing stage in the popular XCMS-based data processing pipeline in lipidomics to improve the removal of artefacts, and to produce putative profiles enabling the statistical analysis of LC/MS datasets. New innovations are:

- *A user-friendly configuration process for each module*. The configuration stage is designed for usability/adaptability for new or expert users, including a set of default settings customized for high-res LC/MS and a graphical user and command line interfaces. All widely used pre-processing tools can now be accommodated (Supplementary Methods).
- *Three new filters and a novel metric method added to the clean-up stage*. The new filters add in-source ion fragments, isotopes and salt clusters to the already broad list of common artefacts that LipidFinder targets and removes from MS datasets. Although isotope annotation can also be performed with CAMERA (included in XCMS), we found that its approach is not tailored optimally for lipidomics, thus we have implemented a new method with a more accurate intensity ratio check. Last, we have introduced a novel FDR method that serves as a metric to assess data quality. This is a new innovation not available in other lipidomics pipelines.
- *A comprehensive re-design of the profiling step to extend its applicability*. Here, the putative lipid profiling stage now returns lipid bulk structures rather than fully annotated, featuring three lipidomics databases from LIPID MAPS. It also returns lipid category scatter plots and fully annotated output files. All these are described in full (Supplementary Data).

## 3. Implementation

LipidFinder 2.0 is fully implemented in Python, supporting versions 2.7 and 3.3 or newer. The source code has been reorganized in a structure more similar to a Python library than a software tool, providing the scripts for the different stages shown in Section 2 and in Supplementary Results. The purpose is to encourage users with experience in programming to reuse LipidFinder’s filters or even entire stages to create their own tailored pipelines. Special attention was paid to *PeakFilter* to ensure it performs efficiently with large datasets. Also, as byproduct of our collaboration with LIPID MAPS (Fahy *et al.*, 2019), we developed an API that provides direct access to the databases, which has significantly reduced the time cost of *MS Search*. We have also linked LipidFinder on LIPID MAPS with XCMS Online to directly import pre-processed files. An analysis of the efficiency of the new implementation is shown in the Supplementary Results. We have produced the user manual in two formats: a PDF file and a Jupyter notebook. The latter converts it into an interactive cookbook where users can learn how to set up LipidFinder and how to use it.

The configuration files for each stage are now stored in JavaScript object notation (JSON) format, a readable text format that can be opened and modified by any text editor. Every data file involved in LipidFinder’s workflow, including those already provided with its source code, is saved either in comma-separated values (CSV) format (*PeakFilter*, *Amalgamator*) or Microsoft Excel spreadsheet (XLS and XLSX) format (*MS Search*), all of them widely supported by data handling applications.

Finally, we performed analysis with a biological dataset comprising lipids extracted from raw and peritoneal macrophages, in order to demonstrate the significant improvements in data quality and visualization achieved using LipidFinder 2.0, detailed in Supplementary Results.

## Funding

This work was supported by a Wellcome Trust grant for LIPID MAPS [203014/Z/16/Z]; and the European Research Council [LipidArrays]. V.B.O. holds a Royal Society Wolfson Research Merit Award. LIPID MAPS receives sponsorship income from Avanti Polar Lipids, Cayman Chemical Co and Merck.

*Conflict of Interest*: none declared.

## Supplementary Material

btaa856_Supplementary_Data
